# Supplementary material for: Modeling glioblastoma heterogeneity as a dynamic network of cell states
Source: Mol Syst Biol. 2021 Sep 16;17(9):e10105. doi: 10.15252/msb.202010105 (PMC8444284; doi:10.15252/msb.202010105)
Supplement: Supplementary file 5 — Source Data for Figure 3 [file MSB-17-e10105-s001.zip › Figure3A_sourcedata/GSEA_3065/hallmarks_state3.GseaPreranked.1623416274744/HALLMARK_G2M_CHECKPOINT.html]

Details for gene set HALLMARK\_G2M\_CHECKPOINT[GSEA]

|  || Dataset | state3 |
| Phenotype | NoPhenotypeAvailable |
| Upregulated in class | na\_pos |
| GeneSet | HALLMARK\_G2M\_CHECKPOINT |
| Enrichment Score (ES) | 0.38957274 |
| Normalized Enrichment Score (NES) | 1.6464356 |
| Nominal p-value | 0.0 |
| FDR q-value | 0.016432686 |
| FWER p-Value | 0.049 |
Table: GSEA Results Summary

  

Fig 1: Enrichment plot: HALLMARK\_G2M\_CHECKPOINT      
 Profile of the Running ES Score & Positions of GeneSet Members on the Rank Ordered List

  

| PROBE | GENE SYMBOL | GENE\_TITLE | RANK IN GENE LIST | RANK METRIC SCORE | RUNNING ES | CORE ENRICHMENT || 1 | CENPF |  |  | 4 | 0.619 | 0.0306 | Yes |
| 2 | SQLE |  |  | 39 | 0.457 | 0.0495 | Yes |
| 3 | TOP2A |  |  | 42 | 0.448 | 0.0718 | Yes |
| 4 | MKI67 |  |  | 59 | 0.407 | 0.0904 | Yes |
| 5 | HNRNPU |  |  | 71 | 0.373 | 0.1078 | Yes |
| 6 | NASP |  |  | 85 | 0.360 | 0.1243 | Yes |
| 7 | SMC2 |  |  | 125 | 0.322 | 0.1359 | Yes |
| 8 | CENPE |  |  | 126 | 0.321 | 0.1520 | Yes |
| 9 | LBR |  |  | 131 | 0.319 | 0.1675 | Yes |
| 10 | KPNA2 |  |  | 157 | 0.308 | 0.1800 | Yes |
| 11 | SFPQ |  |  | 219 | 0.275 | 0.1867 | Yes |
| 12 | SMC1A |  |  | 231 | 0.271 | 0.1990 | Yes |
| 13 | SMC4 |  |  | 265 | 0.255 | 0.2080 | Yes |
| 14 | NCL |  |  | 333 | 0.234 | 0.2119 | Yes |
| 15 | KNL1 |  |  | 342 | 0.231 | 0.2226 | Yes |
| 16 | XPO1 |  |  | 390 | 0.220 | 0.2281 | Yes |
| 17 | SMARCC1 |  |  | 397 | 0.219 | 0.2384 | Yes |
| 18 | KIF20B |  |  | 411 | 0.216 | 0.2477 | Yes |
| 19 | TMPO |  |  | 420 | 0.214 | 0.2575 | Yes |
| 20 | KIF15 |  |  | 428 | 0.213 | 0.2674 | Yes |
| 21 | KIF11 |  |  | 451 | 0.209 | 0.2753 | Yes |
| 22 | SLC38A1 |  |  | 459 | 0.207 | 0.2849 | Yes |
| 23 | HMMR |  |  | 478 | 0.202 | 0.2929 | Yes |
| 24 | TOP1 |  |  | 501 | 0.199 | 0.3003 | Yes |
| 25 | TPX2 |  |  | 544 | 0.193 | 0.3051 | Yes |
| 26 | ATRX |  |  | 638 | 0.180 | 0.3033 | Yes |
| 27 | KIF23 |  |  | 662 | 0.177 | 0.3095 | Yes |
| 28 | HSPA8 |  |  | 678 | 0.175 | 0.3165 | Yes |
| 29 | BUB1 |  |  | 717 | 0.168 | 0.3205 | Yes |
| 30 | PRPF4B |  |  | 724 | 0.167 | 0.3282 | Yes |
| 31 | SLC12A2 |  |  | 751 | 0.164 | 0.3334 | Yes |
| 32 | CASP8AP2 |  |  | 794 | 0.158 | 0.3364 | Yes |
| 33 | RBL1 |  |  | 844 | 0.153 | 0.3384 | Yes |
| 34 | MAD2L1 |  |  | 846 | 0.153 | 0.3459 | Yes |
| 35 | CDK1 |  |  | 915 | 0.147 | 0.3454 | Yes |
| 36 | NSD2 |  |  | 969 | 0.141 | 0.3462 | Yes |
| 37 | ORC6 |  |  | 1000 | 0.138 | 0.3497 | Yes |
| 38 | LMNB1 |  |  | 1021 | 0.137 | 0.3542 | Yes |
| 39 | NDC80 |  |  | 1087 | 0.131 | 0.3532 | Yes |
| 40 | CHAF1A |  |  | 1106 | 0.129 | 0.3576 | Yes |
| 41 | BRCA2 |  |  | 1122 | 0.128 | 0.3623 | Yes |
| 42 | ILF3 |  |  | 1176 | 0.123 | 0.3623 | Yes |
| 43 | TTK |  |  | 1177 | 0.123 | 0.3685 | Yes |
| 44 | MTF2 |  |  | 1230 | 0.119 | 0.3684 | Yes |
| 45 | POLE |  |  | 1305 | 0.115 | 0.3655 | Yes |
| 46 | POLQ |  |  | 1311 | 0.114 | 0.3707 | Yes |
| 47 | GSPT1 |  |  | 1324 | 0.114 | 0.3750 | Yes |
| 48 | NUSAP1 |  |  | 1338 | 0.113 | 0.3791 | Yes |
| 49 | SRSF10 |  |  | 1345 | 0.112 | 0.3840 | Yes |
| 50 | EWSR1 |  |  | 1354 | 0.111 | 0.3887 | Yes |
| 51 | SLC7A5 |  |  | 1394 | 0.109 | 0.3896 | Yes |
| 52 | PBK |  |  | 1524 | 0.102 | 0.3796 | No |
| 53 | WRN |  |  | 1650 | 0.094 | 0.3698 | No |
| 54 | MEIS1 |  |  | 1663 | 0.093 | 0.3731 | No |
| 55 | TENT4A |  |  | 1696 | 0.092 | 0.3739 | No |
| 56 | SRSF2 |  |  | 1697 | 0.092 | 0.3786 | No |
| 57 | MCM2 |  |  | 1762 | 0.088 | 0.3755 | No |
| 58 | AURKB |  |  | 1838 | 0.084 | 0.3710 | No |
| 59 | ARID4A |  |  | 1907 | 0.081 | 0.3671 | No |
| 60 | MCM3 |  |  | 1918 | 0.080 | 0.3700 | No |
| 61 | SLC7A1 |  |  | 1936 | 0.079 | 0.3719 | No |
| 62 | KIF2C |  |  | 1985 | 0.077 | 0.3702 | No |
| 63 | NOTCH2 |  |  | 1990 | 0.076 | 0.3735 | No |
| 64 | STAG1 |  |  | 2064 | 0.073 | 0.3687 | No |
| 65 | RAD21 |  |  | 2150 | 0.070 | 0.3623 | No |
| 66 | UPF1 |  |  | 2199 | 0.069 | 0.3602 | No |
| 67 | MCM6 |  |  | 2237 | 0.067 | 0.3592 | No |
| 68 | KPNB1 |  |  | 2293 | 0.065 | 0.3560 | No |
| 69 | MYC |  |  | 2324 | 0.064 | 0.3557 | No |
| 70 | PDS5B |  |  | 2342 | 0.063 | 0.3569 | No |
| 71 | MCM5 |  |  | 2344 | 0.063 | 0.3599 | No |
| 72 | SRSF1 |  |  | 2450 | 0.059 | 0.3506 | No |
| 73 | CUL3 |  |  | 2465 | 0.058 | 0.3519 | No |
| 74 | CHEK1 |  |  | 2475 | 0.058 | 0.3538 | No |
| 75 | MEIS2 |  |  | 2506 | 0.057 | 0.3531 | No |
| 76 | BARD1 |  |  | 2514 | 0.056 | 0.3551 | No |
| 77 | CCNA2 |  |  | 2529 | 0.056 | 0.3563 | No |
| 78 | KIF22 |  |  | 2545 | 0.055 | 0.3573 | No |
| 79 | CCNF |  |  | 2588 | 0.054 | 0.3552 | No |
| 80 | SS18 |  |  | 2597 | 0.054 | 0.3569 | No |
| 81 | CDC45 |  |  | 2615 | 0.053 | 0.3576 | No |
| 82 | CCNT1 |  |  | 2644 | 0.052 | 0.3569 | No |
| 83 | HIF1A |  |  | 2661 | 0.051 | 0.3576 | No |
| 84 | RBM14 |  |  | 2672 | 0.051 | 0.3590 | No |
| 85 | RACGAP1 |  |  | 2769 | 0.048 | 0.3502 | No |
| 86 | CUL4A |  |  | 3097 | 0.038 | 0.3140 | No |
| 87 | NUP50 |  |  | 3160 | 0.037 | 0.3086 | No |
| 88 | KIF4A |  |  | 3195 | 0.036 | 0.3064 | No |
| 89 | NUMA1 |  |  | 3232 | 0.035 | 0.3040 | No |
| 90 | CDC7 |  |  | 3339 | 0.032 | 0.2933 | No |
| 91 | CCNB2 |  |  | 3502 | 0.028 | 0.2758 | No |
| 92 | AURKA |  |  | 3503 | 0.028 | 0.2772 | No |
| 93 | CDC27 |  |  | 3508 | 0.028 | 0.2781 | No |
| 94 | ODF2 |  |  | 3547 | 0.027 | 0.2750 | No |
| 95 | E2F3 |  |  | 3553 | 0.027 | 0.2758 | No |
| 96 | NUP98 |  |  | 3616 | 0.025 | 0.2698 | No |
| 97 | CUL5 |  |  | 3629 | 0.025 | 0.2697 | No |
| 98 | RASAL2 |  |  | 3679 | 0.023 | 0.2651 | No |
| 99 | TLE3 |  |  | 3709 | 0.022 | 0.2629 | No |
| 100 | ABL1 |  |  | 3737 | 0.022 | 0.2608 | No |
| 101 | SMAD3 |  |  | 3767 | 0.021 | 0.2585 | No |
| 102 | POLA2 |  |  | 3855 | 0.019 | 0.2493 | No |
| 103 | PRC1 |  |  | 3878 | 0.018 | 0.2476 | No |
| 104 | BUB3 |  |  | 3884 | 0.018 | 0.2479 | No |
| 105 | LIG3 |  |  | 3897 | 0.018 | 0.2474 | No |
| 106 | TNPO2 |  |  | 3948 | 0.016 | 0.2424 | No |
| 107 | E2F1 |  |  | 3983 | 0.016 | 0.2392 | No |
| 108 | CDC6 |  |  | 4028 | 0.014 | 0.2348 | No |
| 109 | CUL1 |  |  | 4057 | 0.014 | 0.2322 | No |
| 110 | CDC25A |  |  | 4065 | 0.013 | 0.2321 | No |
| 111 | TGFB1 |  |  | 4110 | 0.012 | 0.2276 | No |
| 112 | FBXO5 |  |  | 4119 | 0.012 | 0.2272 | No |
| 113 | MAP3K20 |  |  | 4123 | 0.012 | 0.2275 | No |
| 114 | PRIM2 |  |  | 4130 | 0.012 | 0.2274 | No |
| 115 | TACC3 |  |  | 4146 | 0.011 | 0.2262 | No |
| 116 | KIF5B |  |  | 4156 | 0.011 | 0.2257 | No |
| 117 | TROAP |  |  | 4205 | 0.009 | 0.2205 | No |
| 118 | PML |  |  | 4353 | 0.005 | 0.2036 | No |
| 119 | HMGB3 |  |  | 4445 | 0.003 | 0.1932 | No |
| 120 | PRMT5 |  |  | 4610 | -0.002 | 0.1741 | No |
| 121 | HIRA |  |  | 4627 | -0.002 | 0.1724 | No |
| 122 | DDX39A |  |  | 4706 | -0.004 | 0.1635 | No |
| 123 | SYNCRIP |  |  | 4713 | -0.004 | 0.1629 | No |
| 124 | FOXN3 |  |  | 4762 | -0.005 | 0.1576 | No |
| 125 | G3BP1 |  |  | 4777 | -0.005 | 0.1562 | No |
| 126 | DBF4 |  |  | 4926 | -0.009 | 0.1394 | No |
| 127 | EZH2 |  |  | 4952 | -0.010 | 0.1370 | No |
| 128 | UCK2 |  |  | 5042 | -0.012 | 0.1272 | No |
| 129 | PAFAH1B1 |  |  | 5051 | -0.012 | 0.1269 | No |
| 130 | RPA2 |  |  | 5078 | -0.013 | 0.1245 | No |
| 131 | ORC5 |  |  | 5091 | -0.013 | 0.1238 | No |
| 132 | YTHDC1 |  |  | 5158 | -0.015 | 0.1168 | No |
| 133 | PLK1 |  |  | 5235 | -0.017 | 0.1088 | No |
| 134 | DMD |  |  | 5320 | -0.019 | 0.0999 | No |
| 135 | MNAT1 |  |  | 5424 | -0.022 | 0.0890 | No |
| 136 | E2F4 |  |  | 5472 | -0.023 | 0.0847 | No |
| 137 | CENPA |  |  | 5506 | -0.024 | 0.0821 | No |
| 138 | MAPK14 |  |  | 5663 | -0.029 | 0.0653 | No |
| 139 | PURA |  |  | 5674 | -0.029 | 0.0656 | No |
| 140 | CTCF |  |  | 5766 | -0.032 | 0.0566 | No |
| 141 | CDKN1B |  |  | 5783 | -0.032 | 0.0564 | No |
| 142 | CDC20 |  |  | 5818 | -0.033 | 0.0541 | No |
| 143 | KMT5A |  |  | 6083 | -0.042 | 0.0254 | No |
| 144 | ATF5 |  |  | 6238 | -0.048 | 0.0098 | No |
| 145 | NOLC1 |  |  | 6241 | -0.048 | 0.0120 | No |
| 146 | KATNA1 |  |  | 6314 | -0.050 | 0.0061 | No |
| 147 | DKC1 |  |  | 6315 | -0.050 | 0.0086 | No |
| 148 | TRA2B |  |  | 6483 | -0.056 | -0.0081 | No |
| 149 | CKS2 |  |  | 6569 | -0.059 | -0.0150 | No |
| 150 | CHMP1A |  |  | 6597 | -0.061 | -0.0151 | No |
| 151 | HNRNPD |  |  | 6663 | -0.064 | -0.0195 | No |
| 152 | HUS1 |  |  | 6879 | -0.075 | -0.0408 | No |
| 153 | CDC25B |  |  | 6935 | -0.078 | -0.0433 | No |
| 154 | TFDP1 |  |  | 7144 | -0.090 | -0.0631 | No |
| 155 | DR1 |  |  | 7214 | -0.095 | -0.0663 | No |
| 156 | CCND1 |  |  | 7456 | -0.113 | -0.0888 | No |
| 157 | SNRPD1 |  |  | 7463 | -0.113 | -0.0838 | No |
| 158 | GINS2 |  |  | 7545 | -0.122 | -0.0871 | No |
| 159 | SAP30 |  |  | 7564 | -0.123 | -0.0831 | No |
| 160 | CKS1B |  |  | 7569 | -0.124 | -0.0773 | No |
| 161 | RAD23B |  |  | 7601 | -0.128 | -0.0745 | No |
| 162 | CDKN2C |  |  | 7609 | -0.128 | -0.0689 | No |
| 163 | BIRC5 |  |  | 7735 | -0.144 | -0.0762 | No |
| 164 | DTYMK |  |  | 7757 | -0.147 | -0.0713 | No |
| 165 | MYBL2 |  |  | 7771 | -0.148 | -0.0654 | No |
| 166 | STMN1 |  |  | 7808 | -0.153 | -0.0619 | No |
| 167 | CBX1 |  |  | 7995 | -0.184 | -0.0744 | No |
| 168 | CDKN3 |  |  | 7998 | -0.184 | -0.0654 | No |
| 169 | AMD1 |  |  | 8120 | -0.208 | -0.0690 | No |
| 170 | CDK4 |  |  | 8168 | -0.220 | -0.0635 | No |
| 171 | JPT1 |  |  | 8212 | -0.231 | -0.0569 | No |
| 172 | PTTG1 |  |  | 8366 | -0.279 | -0.0607 | No |
| 173 | HMGA1 |  |  | 8519 | -0.346 | -0.0611 | No |
| 174 | ODC1 |  |  | 8576 | -0.385 | -0.0483 | No |
| 175 | HMGN2 |  |  | 8644 | -0.449 | -0.0336 | No |
| 176 | MT2A |  |  | 8650 | -0.456 | -0.0113 | No |
| 177 | UBE2S |  |  | 8655 | -0.458 | 0.0112 | No |
Table: GSEA details [plain text format]

  

Fig 2: HALLMARK\_G2M\_CHECKPOINT: Random ES distribution      
 Gene set null distribution of ES for **HALLMARK\_G2M\_CHECKPOINT**
